# Know your blood: Addressing barriers to preconceptual screening for sickle cell disease

**DOI:** 10.1002/hem3.70194

**Published:** 2025-08-07

**Authors:** Ernel Boadi, Alidor Gaspar, Stephen P. Hibbs

**Affiliations:** ^1^ Faculty of Medicine and Dentistry Queen Mary University of London London UK; ^2^ Hidden Pain Society CIC London UK; ^3^ Wolfson Institute of Population Health Queen Mary University of London London UK

Sickle cell disease (SCD) is an inherited hemoglobin disorder, predominantly affecting people with ancestry from Africa or other regions of endemic malaria. Carrier status is common, but many carriers will be unaware of their genotype and the risk of their children having SCD. Although reproductive choices are deeply personal, many people wish to know their hemoglobin genotype to make informed decisions about family planning.

In this article, we explore barriers to current preconceptual screening, illustrated by the experiences of a healthcare professional (Ernel Boadi) and SCD advocate (Alidor Gaspar). We use the term preconceptual broadly, indicating testing at any time before a person has conceived a child—often long before they are considering conceiving children at all. We then suggest avenues to improve access to testing, based on conventional or new testing strategies. Although we focus on SCD carrier testing within the UK National Health Service (NHS), many of these considerations will be relevant to other hemoglobinopathies and within other health systems.

## BARRIERS TO TESTING: PERSPECTIVE FROM A HEALTHCARE PROFESSIONAL (ERNEL BOADI)

My own journey to find out my hemoglobin genotype highlights many of the challenges people face today when trying to get tested. As someone of sub‐Saharan African descent, I have always been interested in knowing my genotype—especially for future family planning. Working in the UK NHS has made it somewhat easier to navigate the system and speak to the right people about screening, but even then, the process has not been straightforward.

Each time I inquired, I was given a random time and day to come in for testing—often during a weekday when I would have to take time off work to travel to a hospital. Despite having an interest in this for years, I still have not managed to get tested. The reality is, since I am not planning to have children anytime soon, it never feels urgent enough to justify the inconvenience. And I know I am not alone in this—accessibility and timing are major barriers that prevent many people from getting screened.

A further problem for people I speak to is a lack of awareness or misinformation. Many simply do not consider genetic risks unless they have a direct family connection to the condition. Some hold a misconception that not having a family member with SCD means that they cannot be a carrier, leading to individuals overlooking screening until it becomes immediately relevant—often during pregnancy.

Finally, cultural and religious beliefs also play a role. Some faiths and traditions emphasize having children naturally with minimal medical intervention, meaning genetic testing can be frowned upon. Others take a flexible, hands‐off approach to family planning, adopting an “if it happens, it happens” attitude—making seeking screening even less likely.

## BARRIERS TO TESTING: PERSPECTIVE FROM A SCD ADVOCATE (ALIDOR GASPAR)

I had the trauma of growing up in a single‐parent home with my mum having sickle cell and me having sickle cell. We have both been in hospital at the same time, and I have had to leave my ward to go and visit her in her ward. At that time, I did not understand how traumatic it was until I grew up and understood what we had gone through. And from then I told myself I would never pass down sickle cell to my child. When I met my wife, if I wanted to continue with our relationship and go on to get married, I told her that I needed the test done. That is how serious I think everyone should be, whether we have SCD or not.

When I do things like workshops, advocacy work, and raising awareness, I try my best to let others know that they must know their genotype. An action off the back of my advocacy work is people going to their primary care doctors or hospitals and asking if they can get a genotype test, so that they can plan for the future. Unfortunately, they are often met with “we can't do that.” Some people resort to saying “my partner has sickle cell, and I want to have a child” to try and access testing, and even then, only some get accepted to test their genotype. Accessible testing is critical to empower individuals and to lower the risks of sickle cell being passed on to the next generation.

## MAPPING A WAY FORWARD

How can we improve upon the current barriers to testing? Some countries take a radical approach by adopting universal testing during adolescence or as a requirement before marriage. Although this would significantly increase access and uptake, it is inappropriate in many cultures and health systems, especially where there is a history of coercive use of medical power against minoritized groups. Increasing access to testing is not the same thing as forcing individuals to know their hemoglobin status.

A more suitable strategy would be to strengthen existing testing pathways within health centers and to understand why individuals are being turned away. Screening on request is available on the UK NHS, but some frontline clinicians may be unaware of this. There may also be a problem of language. Individuals may ask to know their “blood type,” which a clinician will likely understand as relating to blood group antigen systems, for which testing is not routinely funded. Others may request to know their “genotype,” but clinicians may understand this to be different to a “hemoglobinopathy screen,” the common name of the test on many laboratory request systems. Education to frontline clinical staff may partially address such barriers, but because primary care facilities are so numerous and staff turnover is high, such intervention will have limited impact.

Even if individuals seeking testing can access this in health centers, there remain significant barriers to uptake. It takes substantial commitment to book a blood test appointment within inflexible hours, to undergo venepuncture, and to come back for results after days or weeks. To address these barriers, it is worth considering the potential role of point‐of‐care testing (POCT).

POCT for hemoglobinopathies is now accurate, fast, and relatively low‐cost. Some devices, such as Gazelle (Hemex Health, Portland, OR, USA), are able to identify hemoglobin S, C, and E variants with 100% accuracy, alongside variants of clinical significance in addition to Hb S, including beta‐thalassemia trait.^1^ The use of such devices has been predominantly explored in neonatal screening settings.^2^

POCT devices could address many barriers in preconceptual testing. These devices can be brought to community outreach events, rather than requiring individuals to attend primary health centers. They partially mitigate needle‐phobia, as individuals provide a finger‐prick sample rather than undergoing venous puncture. Finally, there is minimal delay between testing and results, with most POCT devices processing samples in around 10 min.

However, the most difficult aspect to address using a community POCT approach may be counseling. Although taking finger‐prick samples and using POCT devices is relatively straightforward, the result provided may have significant implications for an individual and raise many questions, such as partner testing or access to in vitro fertilization (IVF). Trained counselors would either need to be present alongside POCT testing in the community or accessible through robust referral pathways for individuals found to carry a significant hemoglobin variant.

Both strategies can be pursued in parallel. For people seeking to know their status and sickle cell advocates, we suggest the language of “sickle cell carrier testing” rather than “blood group” or “genotype” which can potentially be misunderstood. Simultaneously, community POCT testing should be explored through collaborative codesign involving sickle cell advocates, genetic counselors, and POCT experts. It is time to address frustrating barriers to testing, to support individuals who wish to learn their carrier status, and to enable informed reproductive choices.

## AUTHOR CONTRIBUTIONS

**Ernel Boadi**: Conceptualization; writing—review and editing; writing—original draft. **Alidor Gaspar**: Conceptualization; writing—review and editing; writing—original draft. **Stephen P. Hibbs**: Conceptualization; writing—review and editing; writing—original draft; supervision.

## CONFLICT OF INTEREST STATEMENT

The authors declare no conflicts of interest.

## FUNDING

S.P.H. is supported by a HARP doctoral research fellowship, funded by the Wellcome Trust (Grant number 223500/Z/21/Z).

## Data Availability

Data sharing is not applicable to this article as no datasets were generated or analyzed during the current study.
